# 2724. Community-acquired Pneumonia in Immunocompromised Patients: Predictors and Outcomes Associated with Empiric Broad-spectrum Antibiotic Treatment

**DOI:** 10.1093/ofid/ofad500.2335

**Published:** 2023-11-27

**Authors:** Louis Saravolatz, Tejal N Gandhi, Valerie Vaughn, David Ratz, Jennifer K Horowitz, Ashwin Gupta, Elizabeth McLaughlin, Allison J Weinmann, David Paje, Anurag Malani, Lisa E Dumkow, Scott A Flanders, Lindsay A Petty

**Affiliations:** Michigan Medicine, Ann Arbor, Michigan; Michigan Medicine, Ann Arbor, Michigan; University of Utah Medical School, Salt Lake City, Utah; VA Ann Arbor Health Care, Ann Arbor, Michigan; Michigan Medicine, Ann Arbor, Michigan; Michigan Medicine, Ann Arbor, Michigan; University of Michigan, Michigan Medicine, Ann Arbor, Michigan; Henry Ford Hospital, Detroit, Michigan; University of Michigan, Ann Arbor, Michigan; Trinity Health Michigan, Ann Arbor, Michigan; Trinity Health Grand Rapids, Grand Rapids, Michigan; University of Michigan, Ann Arbor, Michigan; University of Michigan, Ann Arbor, Michigan

## Abstract

**Background:**

Immunocompromised patients (ICP) are at higher risk of infection, but are often excluded from trials for community-acquired pneumonia (CAP). Due to limited data on CAP treatment in ICP, the IDSA/ATS CAP guideline omits recommendations for ICP. We aimed to assess predictors and outcomes associated with empiric broad-spectrum antibiotic (BSA) use versus standard CAP treatment in ICP hospitalized with CAP without IDSA risk factors for drug-resistant organisms (DRO).

**Methods:**

We collected data from non-ICU adult ICP hospitalized with CAP [Table 1] between 1/2017 to 12/2022 at 69 Michigan hospitals who received either BSA on hospital day 1 or 2 or a standard CAP regimen. BSA included anti-MRSA and anti-Pseudomonal antibiotics. We excluded patients with moderate/severe COPD, structural lung disease, pulmonary complications, and IDSA risk factors for DRO. Collected data included patient characteristics, laboratory results, antibiotic data, and 30-day outcomes via phone follow up. Logistic regression was used to assess predictors of empiric BSA accounting for hospital-level clustering. We assessed the association of empiric BSA vs. empiric standard CAP treatment with 30-day outcomes using regression models adjusted for variables associated with probability of treatment and known predictors of each outcome.

**Figure:**
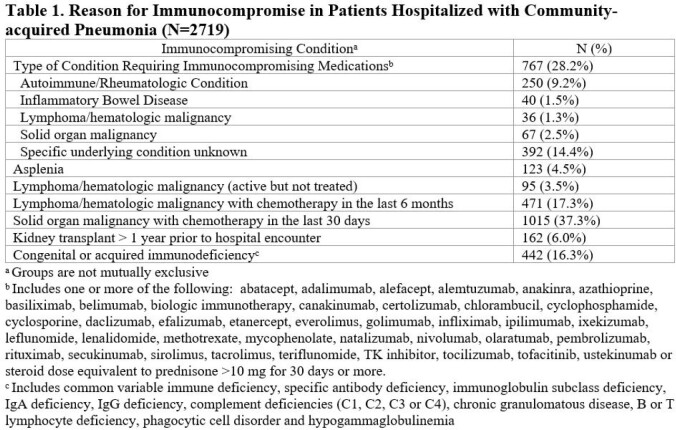

**Results:**

Of 40,882 patients with CAP, 2719 were ICP. Of 2719 ICP with CAP, 41% (N=1115) received standard empiric CAP coverage. Compared to ICP who received standard empiric CAP coverage, those who received empiric BSA had a higher incidence of severe sepsis, higher Pneumonia Severity Index, and hospitalization or IV antibiotics in prior 90 days [Table 2]. The most common CAP pathogen identified was *Pseudomonas* (2.0% (22/1115) standard CAP vs 2.5% (40/1604) BSA) [Table 3]. Of ICP started on BSA, median duration of BSA was 4.4 days [SD 3.4]. After adjustments, receipt of empiric BSA was associated with increased readmission, transfer to ICU and longer hospitalization compared to standard empiric CAP coverage [Table 4].

**Figure:**
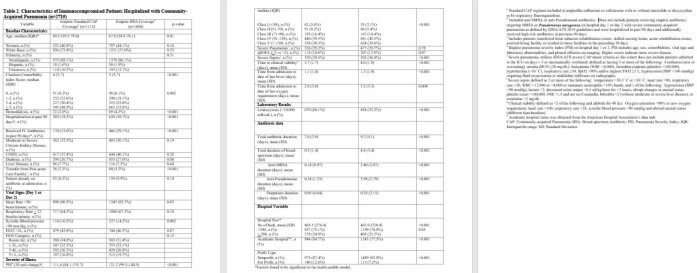

**Figure:**
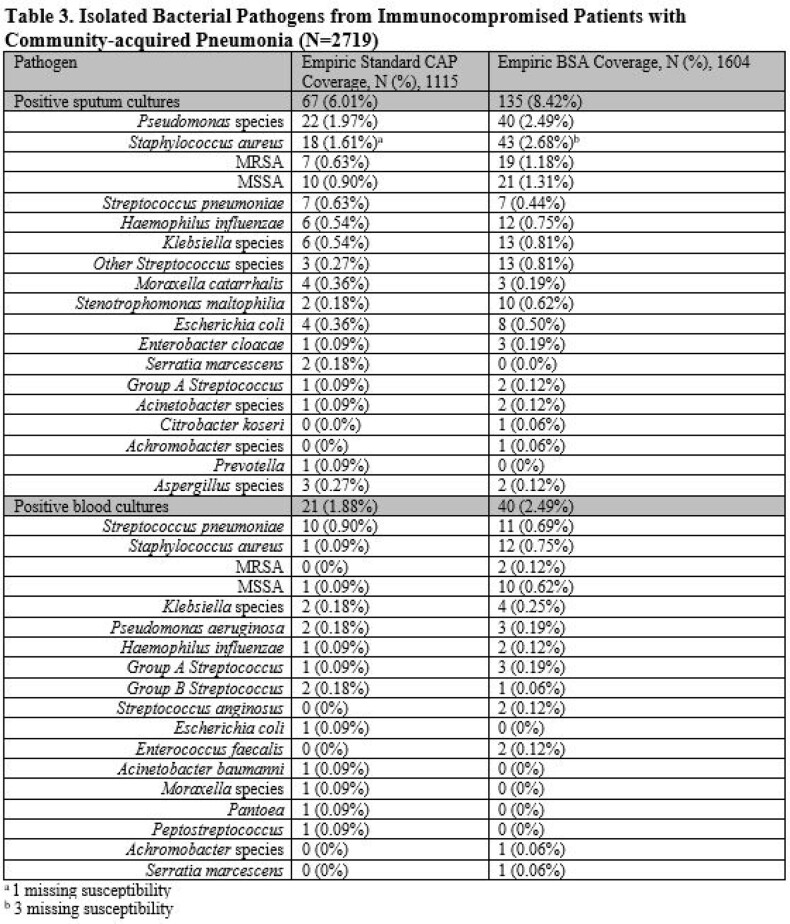

**Figure:**
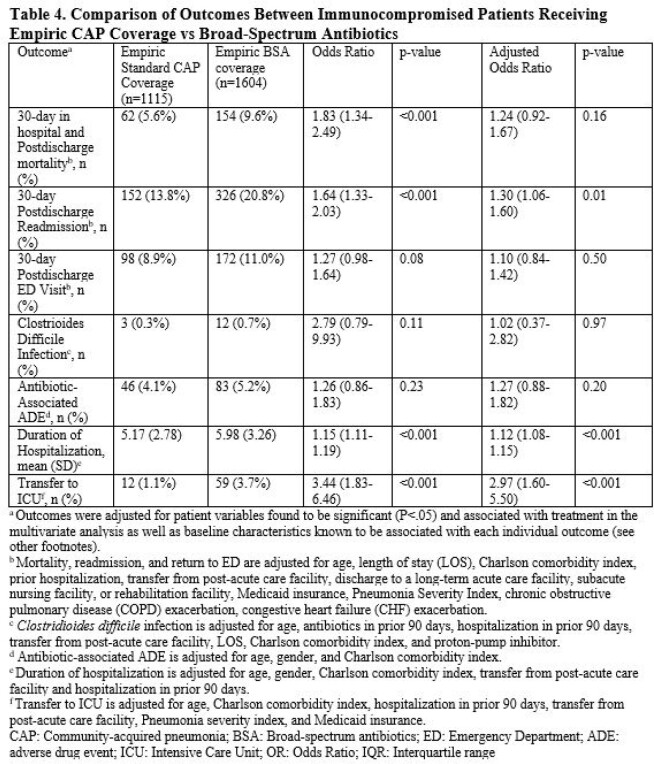

**Conclusion:**

ICP hospitalized with CAP often received empiric BSA but few had identified DRO. Empiric BSA use for CAP in ICP without IDSA risk factors for DRO was associated with worse patient outcomes compared to standard empiric CAP coverage.

**Disclosures:**

**Elizabeth McLaughlin, MS, RN**, Blue Cross Blue Shield of Michigan: Support provided through the Value Partnership Program

